# Production of methane and ethylene from plastic in the environment

**DOI:** 10.1371/journal.pone.0200574

**Published:** 2018-08-01

**Authors:** Sarah-Jeanne Royer, Sara Ferrón, Samuel T. Wilson, David M. Karl

**Affiliations:** Daniel K. Inouye Center for Microbial Oceanography: Research and Education, School of Ocean and Earth Science & Technology, University of Hawaii at Manoa, Honolulu, Hawaii, United States of America; University of Delhi, INDIA

## Abstract

Mass production of plastics started nearly 70 years ago and the production rate is expected to double over the next two decades. While serving many applications because of their durability, stability and low cost, plastics have deleterious effects on the environment. Plastic is known to release a variety of chemicals during degradation, which has a negative impact on biota. Here, we show that the most commonly used plastics produce two greenhouse gases, methane and ethylene, when exposed to ambient solar radiation. Polyethylene, which is the most produced and discarded synthetic polymer globally, is the most prolific emitter of both gases. We demonstrate that the production of trace gases from virgin low-density polyethylene increase with time, with rates at the end of a 212-day incubation of 5.8 nmol g^-1^ d^-1^ of methane, 14.5 nmol g^-1^ d^-1^ of ethylene, 3.9 nmol g^-1^ d^-1^ of ethane and 9.7 nmol g^-1^ d^-1^ of propylene. Environmentally aged plastics incubated in water for at least 152 days also produced hydrocarbon gases. In addition, low-density polyethylene emits these gases when incubated in air at rates ~2 times and ~76 times higher than when incubated in water for methane and ethylene, respectively. Our results show that plastics represent a heretofore unrecognized source of climate-relevant trace gases that are expected to increase as more plastic is produced and accumulated in the environment.

## Introduction

Over the past 50 years, polymer manufacturing has accelerated, from 2x10^6^ metric tonnes (Mt) per year in 1950 to 381x10^6^ Mt per year in 2015, and is expected to double in the next 20 years [1]. The total global production of plastics to date is estimated at 8300x10^6^ Mt, with polyethylene being the most common polymer [2,3], accounting for approximately 36% of all plastic types [1]. In the environment, plastics are vulnerable to weathering and degradation processes, caused by environmental factors such as light, heat, moisture, chemical oxidation and biological activity that are responsible for physical and chemical changes in the structure of the polymer [4].

Polyethylene, like other plastics, is not inert and is known to release additives and other degradation products into the environment throughout its lifetime. For example, the additive bisphenol-A used in the manufacture of many plastic products [5] is leached as plastics age, and hydrocarbon gases are produced during high-temperature decomposition (>202°C) [6]. These chemicals vary amongst different types of plastic and, once released, some can be toxic and have adverse effects on the environment and human health [7–9]. Degradation processes not only affect the chemical integrity of the plastic but also ultimately results in the fragmentation of the polymer into smaller units increasing the surface area exposed to the elements.

Most plastic is synthesized from natural gases [10] and leaching is expected to occur during the aging processes. However, to the best of our knowledge, no previous study has reported hydrocarbon gas emissions from plastics under natural conditions. This study seeks to investigate this phenomenon and its potential environmental consequences.

Our research investigated the production of hydrocarbon gases from polyethylene and other plastics at ambient temperature, with an emphasis on methane (CH_4_), one of the most potent atmospheric greenhouse gases [11–13] and ethylene (C_2_H_4_), which reacts with OH in the atmosphere and increases carbon monoxide concentrations [14,15]. Given the substantial rise in plastic production worldwide, understanding the extent of CH_4_ and C_2_H_4_ emissions from plastic is essential. In addition, we report production rates of ethane (C_2_H_6_), the second most abundant hydrocarbon in the atmosphere after CH_4_, known to enhance the level of tropospheric ozone and carbon monoxide [14,16], and propylene (C_3_H_6_), also a hydrocarbon pollutant in the atmosphere [17]. Since plastics come in different compositions and morphologies, we conducted a series of experiments to evaluate gas production under a variety of environmental conditions. We show that solar radiation initiates the production of these gases for the polymers tested.

## Materials and methods

### Experimental design

Multiple experiments were conducted to assess the relative contribution of plastic in hydrocarbon gas emissions. To assess if different types of plastic emit hydrocarbon gases under natural conditions and the magnitude of these emissions, we tested some of the most common plastics produced worldwide. After selecting the plastic category emitting the greatest amount of hydrocarbon gases, we tested the effect of a change in density and morphology (i.e. pellets, flakes, powder) on hydrocarbon gas emissions. Environmental factors, including solar radiation, UVB (ultraviolet B; 280–320 nm) and solar radiation exposure history were varied as part of this study. These tests were executed in parallel with two long-term experiments assessing the effect of aging for 212 days using virgin pellets and for 152 days using marine plastic from the ocean. Finally, to determine the impact of medium on plastic degradation and gas emissions, we tested the production of gases from plastic incubated in either air or in water. All experiments involving solar radiation were conducted on the roof of C-MORE Hale located in Hawaii, at 21.3°N, 157.9°W.

#### Hydrocarbon gas production from plastics

Initial tests were conducted using different substrates including polycarbonate (PC), acrylic (AC), polypropylene (PP), polyethylene terephthalate (PET), polystyrene (PS), high-density polyethylene (HDPE) and low-density polyethylene (LDPE). Each plastic type was incubated in seawater and exposed to ambient solar radiation for several days, to measure the release of CH_4_ and C_2_H_4_ during incubation. Plastic pieces measured 5.5 x 0.8 cm with a thickness of 0.3 cm and weighed approximately 1.5 g. Each piece of plastic was incubated separately in 30 mL gas-tight cylindrical quartz vials (9.6 cm long by 2.0 cm diameter) filled with seawater filtered using a cartridge with a 0.2 μm pore size. The seawater was collected at 25 m depth at Station ALOHA, a long-term monitoring site located 100 km north of Oahu (Hawaii) in the North Pacific Subtropical Gyre (22.8°N, 158.0°W). The cruise was part of the Hawaii Ocean Time-series (HOT) program (http://hahana.soest.hawaii.edu/hot/) [18] and because it occurred in international waters, no special permission was necessary to sample seawater. The field studies did not involve endangered or protected species. The vials were incubated in an open, shallow (~15 cm depth) water bath maintained at 26°C. For each plastic type, triplicate vials were exposed to ambient solar radiation and another set of triplicates was incubated in the dark (by wrapping the vials with aluminium foil), for a period of six and seven days, respectively. Triplicate quartz vials filled with filtered seawater but without plastic were incubated in light and dark conditions as control treatments. PS was tested in a separate experiment using the same vials, with pieces that were triangular in shape (approximately 5.0 cm perimeter), a thickness of 0.3 cm and a weight of approximately 0.2 g. A set of triplicate vials with no plastic served as control.

#### Effects of solar radiation exposure and ultraviolet radiation on hydrocarbon gas production

Experiments were conducted to determine the effects of aging and irradiance spectrum on hydrocarbon gas production. For these experiments, we used virgin LDPE pellets (Marlex 1017, Chevron Phillips) with a diameter of approximately 5 mm and a density of 0.917 g cm^-3^. Replicate 30 mL quartz vials (n = 4) containing approximately 0.2 g of LDPE pellets in MilliQ water were incubated on the roof of C-MORE Hale and the outer surfaces of the vials were cleaned and shaken weekly. Hydrocarbon gas concentrations were measured approximately every two weeks over a 212-day period. After each measurement, the inner and outer surfaces of the vials were cleaned, and the vials filled with fresh MilliQ water, the previously-exposed LDPE pellets added, and incubated again.

To determine the effect of UV radiation on hydrocarbon gas production, triplicate vials containing 3.0 g of LDPE pellets immersed in MilliQ water were incubated for seven days either in gas-tight 40 mL Pyrex® bottles (11.8 cm long and 2.1 cm in diameter) or in 30 mL quartz vials, after which the gas concentrations were measured. The dark treatment consisted of virgin LDPE pellets with MilliQ water in vials wrapped in aluminium foil. The Pyrex® bottles were scanned using a spectrophotometer and did not transmit UVB radiation (280–320 nm) and hence could help in deciphering the importance of shorter wavelengths for photo-degradation processes.

To determine whether hydrocarbon gas production continues once the plastic is removed from sunlight, triplicate vials containing 3.0 g of LDPE pellets were incubated in 40 mL serum vials in the dark for 14 days at 25°C. The LDPE pellets had been exposed to light for 270 days and 15 days with a control treatment of virgin pellets that had never been exposed to natural light.

#### Effects of polymer morphology and density

An additional test to determine the effect of polymer morphology on hydrocarbon gas production used six different types of polyethylene. Five of the samples were LDPE with three of the samples in pellet form while the other two were either powder or flake. The remaining sample was linear low-density polyethylene (LLDPE) with a density very close to one of the LDPE pellet treatments. For each treatment, 0.4 g of plastic was incubated in gas-tight quartz vials in MilliQ water and exposed to ambient solar radiation for eight days on the roof of C-MORE Hale. For all experiments, the control treatment (n = 3) consisted of vials with MilliQ water but without polyethylene.

#### Production of hydrocarbon gases from environmental plastic debris

In addition to virgin plastic, marine plastic debris from the ocean was collected at Station ALOHA. Plastic pieces were collected using a specialized manta trawl [19] skimming the surface of the ocean for 30 min. The collected plastic (n = 4) was cleaned by soaking in 10% hydrochloric acid for 2 hours and incubated in MilliQ water on the roof of C-MORE Hale as separate pieces (with a weight of approximately 0.2 g) for a period of 152 days. Gas measurements were made every two weeks after which the inner and outer surfaces of the vials were cleaned, filled with MilliQ water, the previously-exposed plastic added, and incubated again. Vials with MilliQ water and without plastic were used as control treatment for each time point.

#### Emission of hydrocarbon gases by LDPE exposed to natural sunlight in air

To determine if LDPE virgin pellets also emit hydrocarbons when exposed directly to air and if this production differs under dark conditions, a pre-weighed amount of virgin LDPE pellets (3.0 g) was placed in 30 mL quartz vials that were flushed with zero-CH_4_ air for 20 seconds and immediately closed. The vials were either exposed to full sunlight or wrapped in aluminium foil. An extra set of flushed vials (without LDPE pellets) for each treatment was also incubated and used as control treatments. At each time point (1, 3, 9 and 17 days), three samples from each treatment (control, light and dark) were removed and analyzed for CH_4_ and C_2_H_4_ concentrations in the headspace.

### Plastic analysis

To identify the type of plastic collected in the ocean, spectra were obtained using a micro-Raman RXN system (Kaiser Optical Systems Inc.) as previously described [20]. A 785 nm laser excitation and a Leica automated microscope stage with a 50 μm slit width were used to determine the micro-Raman spectra of the different plastic types. The environmental samples were compared with known plastics and the spectra analysed using GRAMS/AI™ Spectroscopy software.

### Hydrocarbon analysis

The dissolved gas concentrations were quantified using a purge and trap system [21] connected to a gas chromatograph (Agilent 7890A) equipped with a flame ionization detector. A known volume of sample was transferred from the vials to a sparging chamber using positive pressure supplied by ultra-high purity helium (Airgas) and sparged for 12 min (100 ml min^-1^). The eluting gas stream was passed through a Nafion dryer and Drierite® and Ascarite® columns in order to remove water vapour and CO_2_ before being cryotrapped on a sample loop containing Poropak Q 80/100 (Sigma-Aldrich) and immersed in liquid nitrogen. The trap was subsequently heated for two min to approximately 90°C and the gas released was injected onto a 30 m x 0.32 mm GS-CarbonPLOT analytical capillary column (J&W Scientific) with a temperature program of 38°C for 1.0 min, 65°C for 1.3 min and 150°C for 2.2 min [22]. Calibration was performed by analysis of compressed gas standards containing 10 ± 2% ppm of CH_4_, C_2_H_4_, C_2_H_6_ and C_3_H_6_ in nitrogen (Scott-Marrin). Set volumes of gas standard were analyzed following the same procedure used for sample analysis [23]. The experimental precision of these measurements, based on the coefficient of variation of ten replicate samples, was within 5% for all gases. The limit of detection, given the sample volume typically analyzed for these experiments, was approximately 0.15 nM for all of the gases. The rate of CH_4_ and C_2_H_4_ production in the control treatments (i.e. vials incubated under the same conditions without plastic) was approximately 5%.

### Statistical analysis

Student’s t-distribution (t-test) was used to determine differences between treatments with a level of significance of P < 0.05.

## Results

Preliminary experiments showed that all of the polymer types tested (PC, AC, PP, PET, PS, HDPE and LDPE) produced measurable quantities of CH_4_ and C_2_H_4_ when exposed to ambient solar radiation (Table 1). The rate of production spanned more than two orders of magnitude between the different types of plastic, from 10 to 4100 pmol g^-1^d^-1^ for CH_4_ and ~ 20 to 5100 pmol g^-1^d^-1^ for C_2_H_4_. The highest production rates for both gases were measured for LDPE. Most of the samples kept in the dark did not produce any gas, and those that did had much lower production rates, reaching 120 pmol CH_4_ g^-1^ d^-1^ and 60 pmol C_2_H_4_ g^-1^ d^-1^ for PS, and 50 pmol CH_4_ g^-1^ d^-1^ and 20 pmol C_2_H_4_ g^-1^ d^-1^ for PET and AC, respectively. Gas production from all other plastic types incubated in the dark was either below the detection limit of the instrument or not significantly different from the control treatment (t-test, P>0.11).

**Table 1:** Mean production rates of CH_4_ and C_2_H_4_ from a variety of plastics incubated in water under ambient solar radiation (light) and dark conditions.

Plastic type	Source	CH_4_ (pmol g^-1^d^-1^)		C_2_H_4_ (pmol g^-1^d^-1^)	
		light	dark	light	dark
**Polycarbonate** **(PC)**	www.amazon.com/dp/B000FP83PO/ref=biss_dp_t_asn	10 ± 2	NS	24 ± 5	NS
**Acrylic** **(AC)**	www.minplastics.biz/acrylic_products.html	30 ± 3	NS	24 ± 1	20 ± 1
**Polypropylene** **(PP)**	www.amazon.com/dp/B000ILG19U/ref=biss_dp_t_asn	170 ± 10	NS	50 ± 1	NS
**Polyethylene** **Terephthalate** **(PET)**	www.amazon.com/dp/B0015H4BIE/ref=biss_dp_t_asn	500 ± 20	50 ± 10	64 ± 11	NS
Polystyrene* (PS)	commercial.owenscorning.com/products/foam/	730 ± 110	120 ± 30	910 ± 10	60 ± 5
**High-density** **Polyethylene** **(HDPE)**	www.amazon.com/dp/B000ILG0TQ/ref=biss_dp_t_asn	90 ± 10	NS	190 ± 20	NS
**Low-density** **Polyethylene** **(LDPE)**	www.amazon.com/dp/B000ILG118/ref=biss_dp_t_asn	4100 ± 200	NS	5100 ± 400	NS

Relevant information regarding the polymer sources is also included. The errors represent the standard deviation of triplicate samples.

NS: final concentrations not significantly different from those in the control treatment (t-test, P>0.05).

*: Polystyrene incubations lasted for 14 days and were conducted in MilliQ water.

**Fig 1:**
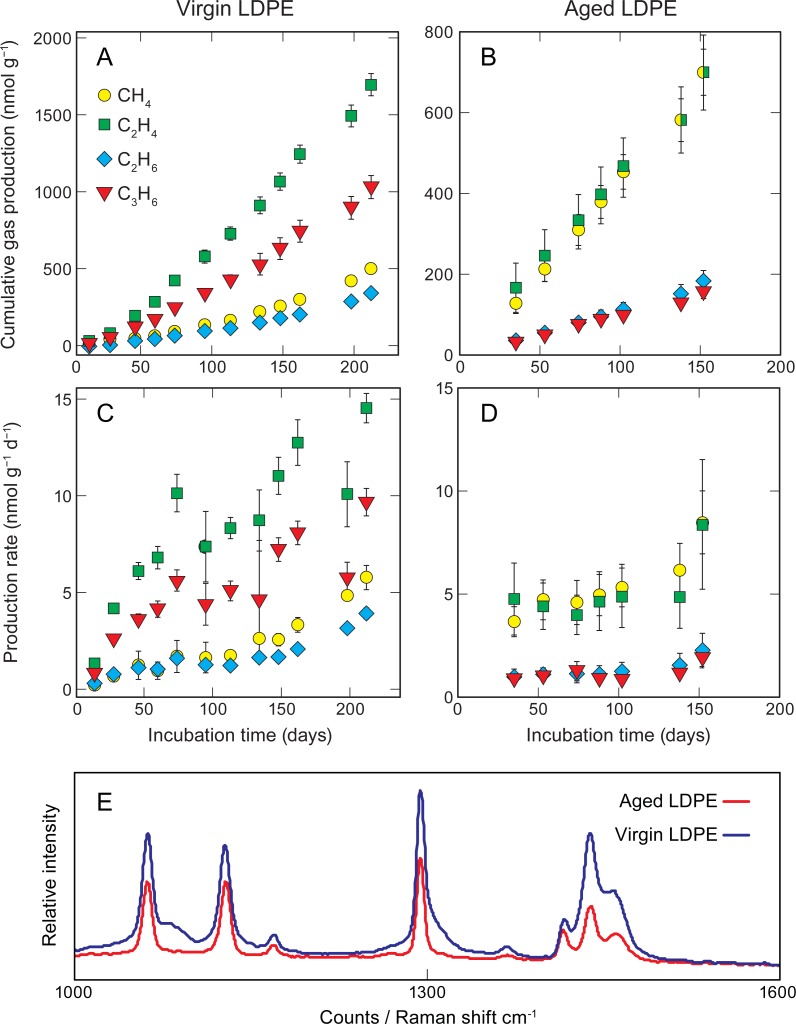
Gas production from LDPE exposed to ambient solar radiation and identification of plastic collected in the open ocean. The cumulative amount of hydrocarbon gases normalized to weight and production rates of CH_4_, C_2_H_4_, C_2_H_6_ and C_3_H_6_ from (A) & (C) virgin LDPE pellets (0.2 g) and (B) & (D) LDPE plastic pieces (0.2 g) collected from the open ocean. Measurements were taken at approximately two-week intervals during the incubation period. Error bars represent the error propagation of the standard deviation of the mean for each time point. Panel (E) represents the micro-Raman spectra for the aged LDPE collected in the open ocean (red) and the virgin LDPE pellets (blue).

The spectra derived from Raman analysis on plastic debris pieces collected from the open ocean were compared with different types of commercially sourced common plastics. The spectra matched with LDPE and therefore the plastic collected from the open ocean was hereafter referred to as “aged LDPE” (Fig 1E). For both types of plastic, the cumulative amount of hydrocarbon gases increased with time although the relative contribution of the gases produced was variable (Fig 1A and 1B). The rates of hydrocarbon gas production are normalized to weight and to convert to surface area, the pellet weight of approximately 35 mg had a surface area ranging from ca. 70 mm^2^ to 315 mm^2^. For the virgin LDPE pellets, the cumulative gas emissions during 212 days were 500, 1700, 340 and 1000 nmol g^-1^ of CH_4_, C_2_H_4,_ C_2_H_6_ and C_3_H_6_, respectively (Fig 1A). For aged plastics, the cumulative gas emissions during 152 days were 700, 700, 180 and 160 nmol g^-1^ of CH_4_, C_2_H_4,_ C_2_H_6_ and C_3_H_6_, respectively (Fig 1B). Gas production rates increased over time for the virgin pellets (Fig 1C) but were relatively constant for the aged plastic (Fig 1D). Except for CH_4_, the weight-normalized gas production rates were lower for the aged plastic compared to virgin pellets. During this period of time (December 2016 to April 2017), the PAR intensity at noon, when the sun was at its zenith, varied from 1496 to 1787 μM m^-2^s^-1^.

**Fig 2:**
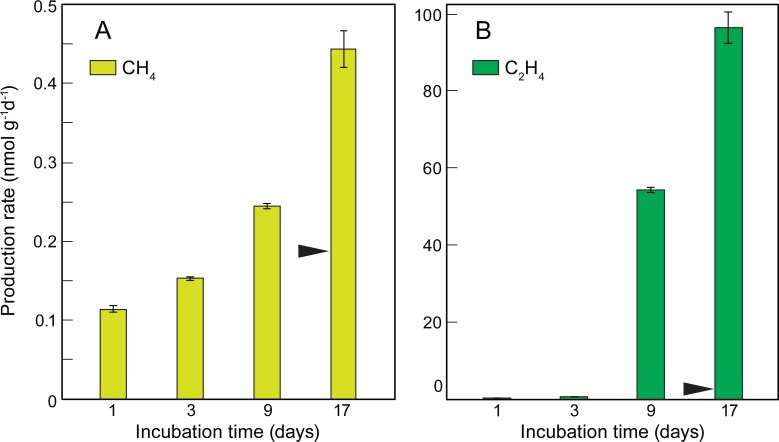
Production rates of CH_4_ and C_2_H_4_ from virgin LDPE pellets exposed to sunlight in air. Mean production rates of CH_4_ (A) and C_2_H_4_ (B) from LDPE virgin pellets incubated in air under ambient solar radiation for 1, 3, 9 and 17 days. The error bars represent the standard deviation of triplicate samples. The emission of CH_4_ in the dark treatment was below the detection limit and less than 0.3 nmol g^-1^d^-1^ for C_2_H_4_. The black arrow indicates the production rate of CH_4_ and C_2_H_4_ from virgin LDPE pellets incubated in MilliQ water for 14 days (Fig 1).

As shown in previous experiments (Fig 1 and Table 1), LDPE releases large amounts of CH_4_, C_2_H_4_, C_2_H_6_ and C_3_H_6_ into seawater and MilliQ water when exposed to sunlight. CH_4_ and C_2_H_4_ production rates from virgin LDPE pellets were larger (for similar incubation periods) when the plastic was exposed to sunlight directly in air (Fig 2), rather than immersed in water (Fig 1). In that case, production rates for both gases increased with time and ranged from 0.11 to 0.44 nmol CH_4_ g^-1^d^-1^ and 0.32 to 96 nmol C_2_H_4_ g^-1^d^-1^ after 17 days of incubation (Fig 2). Those final rates are approximately 2 and 76 times larger than CH_4_ and C_2_H_4_ production from LDPE virgin pellets incubated in MilliQ water for 14 days (Figs 1 and 2). No detectable emissions of any of the gases were observed in the control or dark treatments (data not shown).

**Fig 3:**
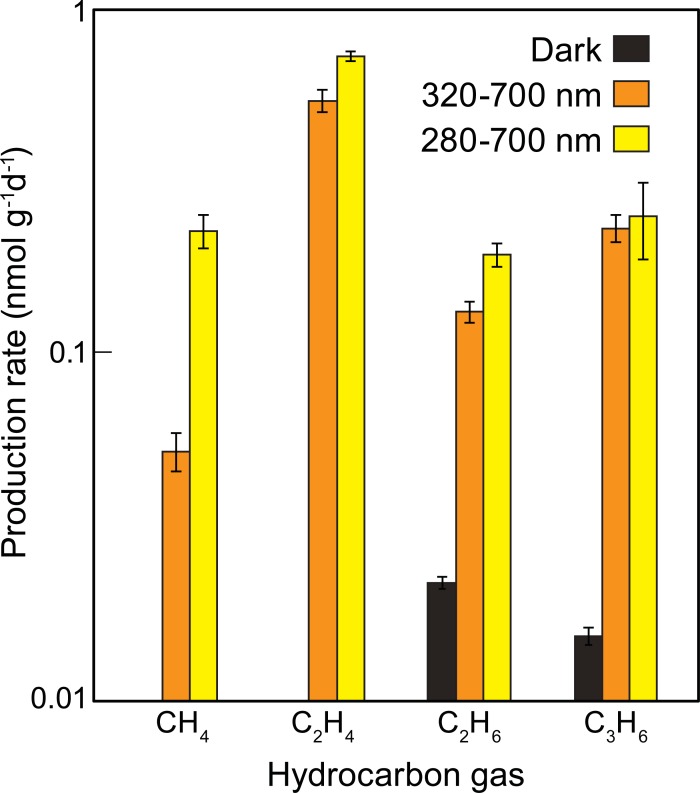
Effect of spectral irradiance on hydrocarbon gas production from LDPE. Production rates of hydrocarbon gases (CH_4_, C_2_H_4_, C_2_H_6_ and C_3_H_6_) from virgin LDPE pellets kept in the dark, exposed to full solar radiation (280–700 nm) or to solar radiation filtered for UVB (320–700 nm). Error bars represent the standard deviation of triplicate samples.

The emissions of hydrocarbon gases were measured using virgin LDPE pellets exposed for seven days to the full solar spectrum (FULL; 280–700 nm) and to solar radiation without UVB ((-)UVB; 320–700 nm; Fig 3). Except for C_3_H_6_ (t-test, P = 0.636), the results showed significant differences (t-test, P<0.002) between the two treatments, with consistently lower rates for the (-)UVB treatment corresponding to 23%, 74%, and 68% of the FULL treatment for CH_4_, C_2_H_4_ and C_2_H_6_, respectively. All dark treatments showed emission rates less than 0.1 nmol g^-1^d^-1^.

LDPE pellets incubated in the dark after exposure to light produced measurable quantities of hydrocarbon gases. The rates of CH_4_ (0.11 nmol g^-1^d^-1^) and C_2_H_4_ (0.52 nmol g^-1^d^-1^) production were equivalent to 55% and 40%, respectively, of the rates when the pellets were incubated in the light (0.20 nmol CH_4_ g^-1^d^-1^ and 1.3 nmol C_2_H_4_ g^-1^d^-1^). In addition, environmentally-aged LDPE plastic collected from the open ocean and incubated for 14 days in the dark under ambient outdoor temperature [18.5–32.5°C] also produced 0.37 ± 0.11, 0.14 ± 0.09, 0.21 ± 0.12 and 0.06 ± 0.03 nmol g^-1^d^-1^ of CH_4_, C_2_H_4,_ C_2_H_6,_ and C_3_H_6_, respectively. Pellets that had been previously exposed to 270 days of natural solar radiation and incubated at 25°C were amongst the highest production rates for all gases with 0.23 ± 0.02, 0.41 ± 0.05, 2.6 ± 0.22 and 0.70 ± 0.09 nmol g^-1^d^-1^ of CH_4_, C_2_H_4,_ C_2_H_6,_ and C_3_H_6_, respectively. These results indicate that once initiated, the production of hydrocarbon gases continues in the dark.

Morphology has an important effect on hydrocarbon gas production. LDPE powder exhibited weight-normalized CH_4_ and C_2_H_4_ production rates that were 488 and 132 times higher than LDPE pellets, respectively (Table 2). All forms of LDPE, except LLDPE, produced measurable quantities of hydrocarbon gases with CH_4_ and C_2_H_4_ production rates ranging from 0.08 to 55 nmol CH_4_ g^-1^d^-1^ and 0.16 to 21 nmol C_2_H_4_ g^-1^d^-1^. With respect to the LDPE pellets of varying densities, there were significant differences in CH_4_ production between treatments (t-test, P<0.03), with lowest rates for the lowest density treatment and highest rates for the highest density treatment. No other consistent pattern for the other hydrocarbon gases was observed with the different densities. Interestingly, the ratios of the different gases produced varied across LDPE pellet densities. For example, the C_2_H_4_: CH_4_ ratio varied from 1.3 to 4.4 and the C_2_H_4_:C_2_H_6_ ratio varied from 1.9 to 8.6 indicating that degradation processes were variable for the different morphologies under these experimental conditions.

**Table 2:** Effect of polymer morphology and density on hydrocarbon gas production from polyethylene.

Source/Product #	Polyethylene category	Morphology	Density (g cm^-3^)	CH_4_ (nmol g^-1^d^-1^)	C_2_H_4_ (nmol g^-1^d^-1^)	C_2_H_6_ (nmol g^-1^d^-1^)	C_3_H_6_ (nmol g^-1^d^-1^)
Sigma 42078	LLDPE	Pellets	0.918	NS	NS	NS	NS
Marflex 1017	LDPE	Pellets	0.917	0.08 ± 0.02	0.35 ± 0.18	0.19 ± 0.03	0.29 ± 0.10
Marflex 1122	LDPE	Pellets	0.920	0.12 ± 0.02	0.16 ± 0.21	0.07 ± 0.03	0.18 ± 0.14
Sigma 428043	LDPE	Pellets	0.926	0.31 ± 0.04	0.86 ± 0.09	0.10 ± 0.01	0.65 ± 0.06
Sigma 427799	LDPE	Flakes	0.906	2.2 ± 1.1	3.2 ± 1.9	1.3 ± 0.8	2.5 ± 1.3
Sigma 427772	LDPE	Powder	0.920	55 ± 4	21 ± 2	13 ± 1	36 ± 5

Mean production rates of hydrocarbon gases from different polyethylene products of different densities and morphologies exposed to ambient solar radiation for 14 days. Relevant information regarding the polymers is also included in the table: the source and product number, the polyethylene category, the morphology of the product and the density at 25°C. The error represents the standard deviation of triplicate samples.

NS: final concentrations were not significantly different from those in the control treatment (t-test, P>0.05).

## Discussion

### The influence of plastic composition on hydrocarbon gas production

Our results show that different types of plastics, that are commonly used and dispersed in the environment worldwide, produce CH_4_ and C_2_H_4_ under environmental conditions. We hypothesize that the relative amounts of low-molecular-weight hydrocarbon gas molecules that are released from plastic substrates depend on the molecular structure of the plastic including the degree of branching, the addition of plasticizers, as well as the manufacturing process. For example, among the plastic materials tested, LDPE produced the largest amounts of CH_4_ and C_2_H_4_, probably due to its weaker structure and more exposed hydrocarbon branches. In contrast, with a more compact structure, lower permeability and fewer accessible active sites, degradation of HDPE resulted in lower emission.

### Long-term hydrocarbon gas emission from plastics

The release of greenhouse gases from virgin and aged plastic over time indicates that polymers continue to emit gases to the environment for an undetermined period. We attribute the increased emission of hydrocarbon gases with time from the virgin pellets to photo-degradation of the plastic, as well as the formation of a surface layer marked with fractures, micro-cracks and pits [24–26]. With time, these defects increase the surface area available for further photo-chemical degradation and therefore might contribute to an acceleration of the rate of gas production. It is also known that smaller particles of secondary origin termed ‘microplastics’ [27,28] are eventually produced and may further accelerate gas production. The initial shape of the polymer is also a potential factor contributing to the variability in hydrocarbon production because items of the same mass but with different shapes have different surface-to-volume ratios. Small fragments not only have a greater surface-to-volume ratio than larger items, but they also tend to have longer edge lengths relative to their volume [29]. This predicts that in the environment, as plastic particles degrade and become smaller, they will also emit more hydrocarbon gases per unit mass. The emission of gases from the aged plastic collected from the ocean (age unknown at the start of the experiment) indicates that production may continue throughout the entire lifetime of the plastic. We hypothesize that lower production rates from environmentally aged plastic are probably due to the presence of plasticizers that retard photo-degradation by counteracting the negative effect of UV radiation.

### Driving factors for gas emissions

Manipulation of the radiation spectrum showed that UVB increases the production of hydrocarbon gases relative to longer wavelengths, which is consistent with its key role in the photo-oxidative degradation processes for polyethylene [26]. Indeed, solar photon flux, especially in the UV portion of the spectrum, provides the activation energy necessary to initiate degradation due to bond cleavage and depolymerisation, which affects the mechanical properties of the material [26,30,31]. We hypothesize that this process yields hydrocarbon gas molecules that are released into the environment. Although the emission of hydrocarbon gases is enhanced by photo-degradation in the UVB spectrum, our results indicate that UVB radiation is not essential for the initiation or the continuous production of hydrocarbon with time. Once initiated by solar radiation, emission of the gases continues in the dark, at a rate that depends on previous radiation exposure.

### Effects of morphology and density of polyethylene on gas emissions

While the production of hydrocarbon gases was prevalent in LDPE, the different morphologies and densities within LDPE products resulted in a change in the relative concentrations of the different gases. LDPE in powder form, with a much higher surface area than pellets with the same density and weight, produced the highest amount of hydrocarbon gases. Along with morphology and density, the average molecular weight that varies with the polymer chain length and hence the number of branched molecules exposed may also be a controlling factor in hydrocarbon emissions. Unfortunately, this information is proprietary and very rarely available from suppliers. Also, even polyethylene, which is known to be the simplest polyolefin, can form various conformational structures of the macromolecular chains, which in its solid state assume various states of intermolecular order [10]. This heterogeneity within the same type of plastic would affect the production rates of the different hydrocarbon gases and consequently the ratios among them. Yet, due to the heterogeneity of the products even within the same type of plastic, the nature of the reactions is unknown. The shortening of the long polymer chain and its breakdown into smaller units can be caused by different mechanisms such as random chain scission, end-chain scission, chain-stripping or cross-linking [28]. These reactions would lead to the production of hydrocarbon gases including those presented in this study.

## Closing comments

The global production of plastic is large [1] and the amount of plastic waste generated in 2010 from 192 countries was 275x10^6^ Mt from which 4.8x10^6^ to 12.7x10^6^ Mt was estimated to enter the ocean [32]. By 2025, the amount of plastic waste input to marine systems might increase by an order of magnitude if waste management is not improved [32]. Because polyethylene is the most common polymer, it is anticipated to be the most common form of plastic pollution in surface ocean waters worldwide [33–35]. In addition, as microplastics with greater surface area are produced hydrocarbon gas production rates will likely accelerate. The results from this study indicate that hydrocarbon gas production may continue indefinitely throughout the lifetime of plastics. The amount of plastic material in the environment exposed to full sunlight exceeds the quantity of submerged plastic. Our experiments indicated that emissions of hydrocarbon gases are even greater (up to 2 times higher for CH_4_ and up to 76 times higher for C_2_H_4_) in air compared to in water. The difference in emission rate for plastic in water compared to plastic exposed to air is partly due to temperature and heat build-up, [36] resulting in the plastic material reaching a temperature higher than the surrounding medium. Furthermore, plastic exposed to air is subjected to less biofouling than in aquatic environments because of the lack of a fluid medium that facilitates microorganisms settling onto the plastic substrate resulting in more surfaces directly exposed to solar radiation and more conducive to deformation, degradation and hydrocarbon production. Therefore, in warm climates, higher hydrocarbon production rates are expected for plastic exposed to air compared to plastic in aquatic environments.

At this stage, very little is known about the contribution of plastic to CH_4_, C_2_H_4_ and other hydrocarbon gas budgets. This study reports the first measurements of hydrocarbon production by plastic under ambient conditions. Based on the rates measured in this study and the amount of plastic produced worldwide CH_4_ production by plastics is likely to be an insignificant component of the global CH_4_ budget. However, for the other hydrocarbon gases with much lower global emissions to the atmosphere compared to CH_4_, the production from the plastics might have more environmental and global relevance. The fate of these gases is also not well constrained, but sinks are likely to be bacterial oxidation to carbon dioxide with a portion of these gases emitted to the atmosphere. Given the ongoing rate at which plastic is being produced, used and exposed on land and the future trend in mismanaged plastic waste ending up in marine systems [32], the amount of plastic exposed to the environment will likely increase with time and so too will the amount of CH_4_ and C_2_H_4_ emitted from polymers. In addition, degradation of plastics in the environment leads to the formation of microplastics with greater surface area, which may accelerate hydrocarbon gas production. Due to the longevity of plastics and the large amounts of plastic persisting in the environment, questions related to the role of plastic in the CH_4_ and C_2_H_4_ global budgets should be prioritized and addressed by the scientific community.

## Supporting information

S1 Dataset Dataset used for Figs 1, 2 and 3. (XLSX)
